# Supplementary material for: Social stress worsens colitis through β-adrenergic–driven oxidative stress in intestinal mucosal compartments
Source: Brain Behav Immun. Author manuscript; Available in PMC 2026 Feb 26. (PMC12943163; doi:10.1016/j.bbi.2025.106222)

**Caetano-Silva et al.** **– Supplementary material**

**Social stress worsens colitis through β-adrenergic–driven oxidative stress in intestinal mucosal compartments**

**Table S1.** Primers (Forward and Reverse, Integrated DNA Technologies, Coralville, IA) used for Fluidigm analysis.

| **Gene** | **Forward sequence** | **Reverse sequence** |
| --- | --- | --- |
| *Apoe* | GACCCAGCAAATACGCCTG | CATGTCTTCCACTATTGGCTCG |
| *B3galt5* | AGGCTAGTTTACGCCTCCATT | AGGAACTTCCCGTGACTTTTCT |
| *Bcl6* | CCGGCACGCTAGTGATGTT | TGTCTTATGGGCTCTAAACTGCT |
| *Ccnd1* | GCGTACCCTGACACCAATCTC | ACTTGAAGTAAGATACGGAGGGC |
| *Cd55* | ACCCCGGTGCATAGAGAAATC | GGATGACGTACTGTTGTCTTGG |
| *Clca4b* | TGTCACCTGGAGCAAACAAGCC | CCAGGTTGTAAGTCCAAACGCC |
| *Creb3l3* | CAGTCAGCTCAAGAAAGCAGG | TGGTTCTGGGCAGTACACG |
| *Cxcl2* | CGCTGTCAATGCCTGAAGAC | ACACTCAAGCTCTGGATGTTCTTG |
| *Duox2* | ACCCTGGACCTCTATTCAG | ACAGCCCATTCCTAGTGT |
| *Duoxa2* | ACCGCTGCTCATTGTTATCC | AGTGCACAGCCACAATTTCG |
| *F2* | TTCGACCCCGAGGTGAAACT | CCCCGCAACATAGCACCAT |
| *Fut1* | TACCTCATCCATTGCAGACATCT | CTCCTGGGGTGATTGTCCAAG |
| *Fut2* | ACCTCCAGCAACGAATAGTGA | GCCGATGGAATTGATCGTGAA |
| *Gda* | AGGAGTGGTGCTTCAAACCAT | GGGATGAGTCAGTGTGAATTGT |
| *Hc* | GAACAAACCTACGTCATTTCAGC | GTCAACAGTGCCGCGTTTT |
| *Ido1* | GCTTTGCTCTACCACATCCAC | AGCTGCCCGTTCTCAATCAG |
| *Il1b* | GCACTACAGGCTCCGAGATGAAC | TTGTCGTTGCTTGGTTCTCCTTGT |
| *Il6* | GACTGATGCTGGTGACAAC | ATCCTCTGTGAAGTCTCCTC |
| *Irs1* | CTCCTGCTAACATCCACCTTG | AGCTCGCTAACTGAGATAGTCAT |
| *Itgb6* | ATGGGGATTGAGCTGGTCTG | GACAGGTGGGTGAAATTCTCC |
| *Jak3* | CCATCACGTTAGACTTTGCCA | GGCGGAGAATATAGGTGCCTG |
| *Lbp* | TCCATCGGTGTCCGAGGCAAAT | AGGTCCACTGAAATGGTGACACC |
| *Ly6a* | AGGAGGCAGCAGTTATTGTGG | CGTTGACCTTAGTACCCAGGA |
| *Ly6c1* | GCAGTGCTACGAGTGCTATGG | ACTGACGGGTCTTTAGTTTCCTT |
| *Muc1* | GGCATTCGGGCTCCTTTCTT | TGGAGTGGTAGTCGATGCTAAG |
| *Nos2* | AGTGAAAAGTCGAGCCGCA | ACAATCCACAACTCGCTCCA |
| *Ptk6* | CTCAGGCCGTGCGACATTA | GTCTTATGGTAGTCCACAAGCTC |
| *Saa1* | GGAGTCTGGGCTGCTGAGAAAA | TGTCTGTTGGCTTCCTGGTCAG |
| *Saa2* | TGGCTGGAAAGATGGAGACAA | AAAGCTCTCTCTTGCATCACTG |
| *Saa3* | AACTATGATGCTGCCCGGAG | GCTCCATGTCCCGTGAACTT |
| *Sbno2* | TTCGCTGCGCTCAACAAGGA | TGACAGGGAATCCACAGATGAA |
| *Socs3* | ATGGTCACCCACAGCAAGTTT | TCCAGTAGAATCCGCTCTCCT |
| *Sphk1* | ATGGAACCAGTAGAATGCCCT | TCCGTTCGGTGAGTATCAGTTTA |
| *St8sia1* | GCTACCCGTAGGAGCCAGT | CAGCACCCCTTGCACAATCT |
| *Stat3* | GTAGTGCTGCCCCGTACCTG | AGCGACTCAAACTGCCCTCC |
| *Tirap* | ATCTCCCAGGAAAGCCACCTCT | GGTAGGTGACATTCCTGAACTGC |
| *Tlr4* | CGGGAGAATCCTGTGGACAA | CTCAGACTCGGCACTTAGCA |
| *Tnf* | AGGCACTCCCCCAAAAGATG | GTAGACAGAAGAGCGTGGTGG |
| *Tnfrsf1b* | ACACCCTACAAACCGGAACC | AGCCTTCCTGTCATAGTATTCCT |
| *Xdh* | GTTGGTTTCAGCGTCAGGAG | CCCAAGTGGCAGTTTTGAGT |

**Supplementary Figure S1. Catecholamine and corticosterone levels in RST-exposed mice. (A)** Epinephrine, **(B)** norepinephrine, and **(C)** corticosterone concentrations in colonic tissue and **(D)** serum corticosterone levels, following restraint stress (RST), as measured by ELISA. Data are shown as fold change relative to control; p-values from Mann–Whitney test are indicated (n = 5-6/group).


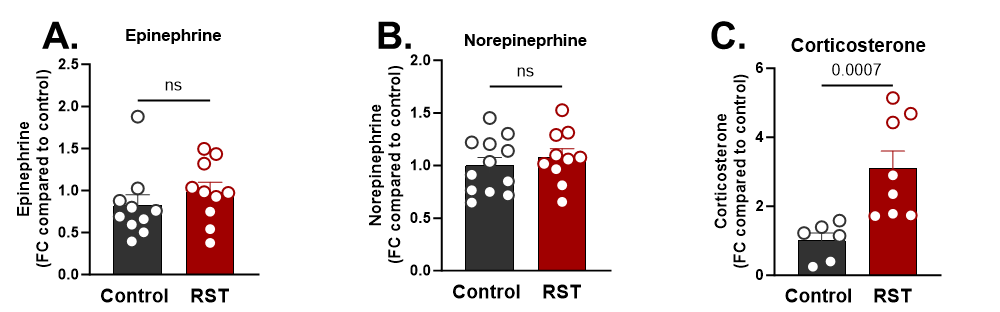


**Supplementary Figure S2. β-adrenergic receptor blockade prevents stress-induced increases in serum LBP and partially attenuates microbiome shifts associated with epithelial ROS/RNS responses. (A)** Serum levels of lipopolysaccharide-binding protein (LBP) were measured by ELISA in mice exposed to social defeat stress (SDR) or left undisturbed, with or without treatment with the β-adrenergic receptor antagonist propranolol. Data were analyzed using one-way ANOVA followed by Tukey’s post hoc test. Statistical significance was set at *p* < 0.05. Values are expressed as mean ± SEM. n=5-6/group. **(B)** Principal coordinates analysis (PCoA) of Bray–Curtis dissimilarity reveals SDR-induced shifts in colonic microbiome beta-diversity. **(C–F)** Alpha-diversity assessed by Chao1 index from SDR mice treated with stress hormone receptor antagonists: **(C)** glucocorticoid receptor (mifepristone), **(D)** corticotropin-releasing factor receptor (antalarmin), **(E)** α₂-adrenergic receptor (idazoxan), and (**F)** β-adrenergic receptor (propranolol). **(G-H)** Heat tree visualizations of differential taxonomic abundance in **(G)** SDR vs. control mice and **(H)** SDR mice treated with propranolol vs. saline.


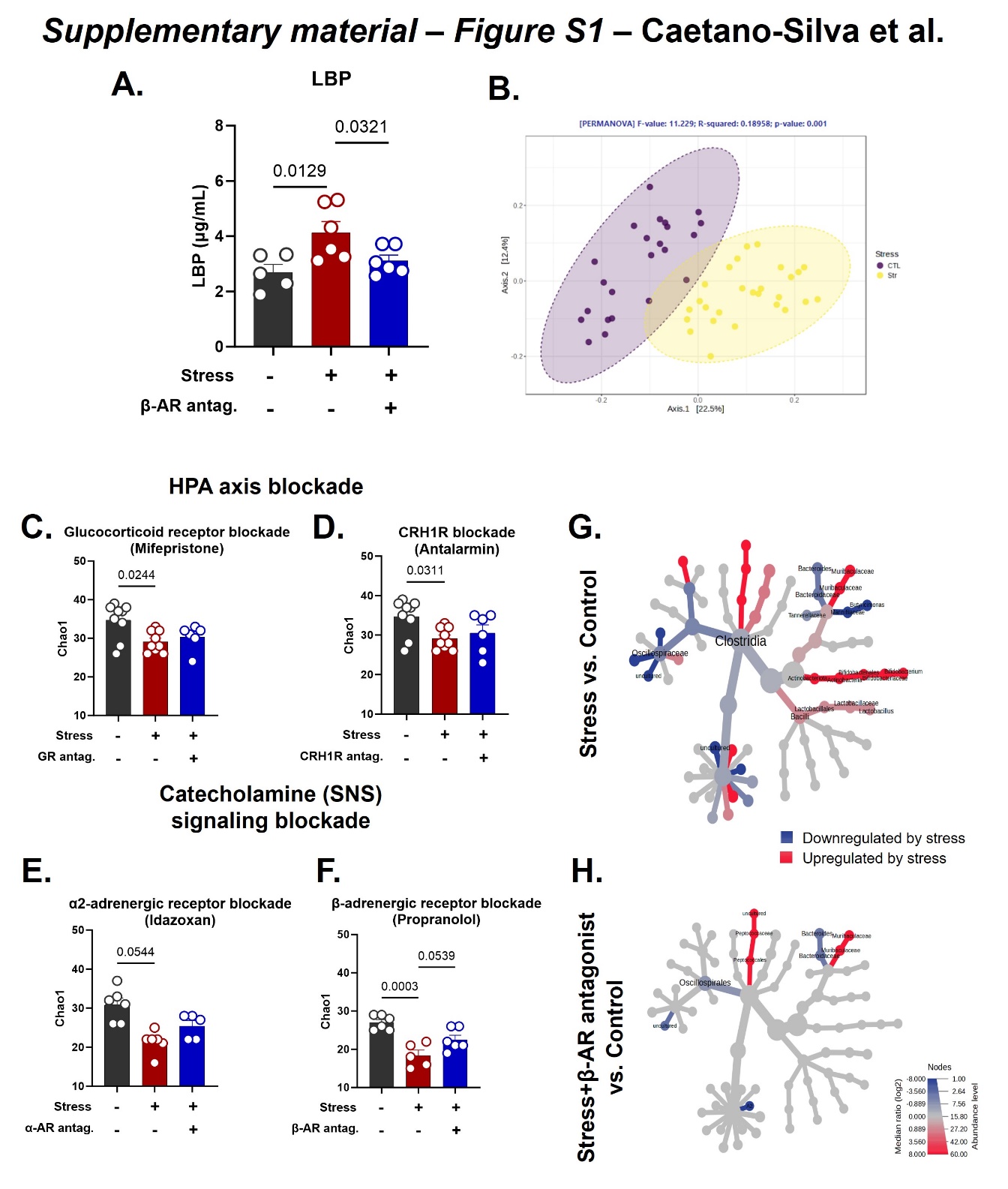


**Supplementary Figure S3. Food intake is not significantly altered by stress or propranolol treatment during *C. rodentium* infection or DSS-induced colitis.** Food intake was monitored daily or every other day in two experimental models: **(A)** *Citrobacter rodentium* infection and **(B)** dextran sulfate sodium (DSS)-induced colitis. Data are presented as mean ± SEM (n = 5-6/group).


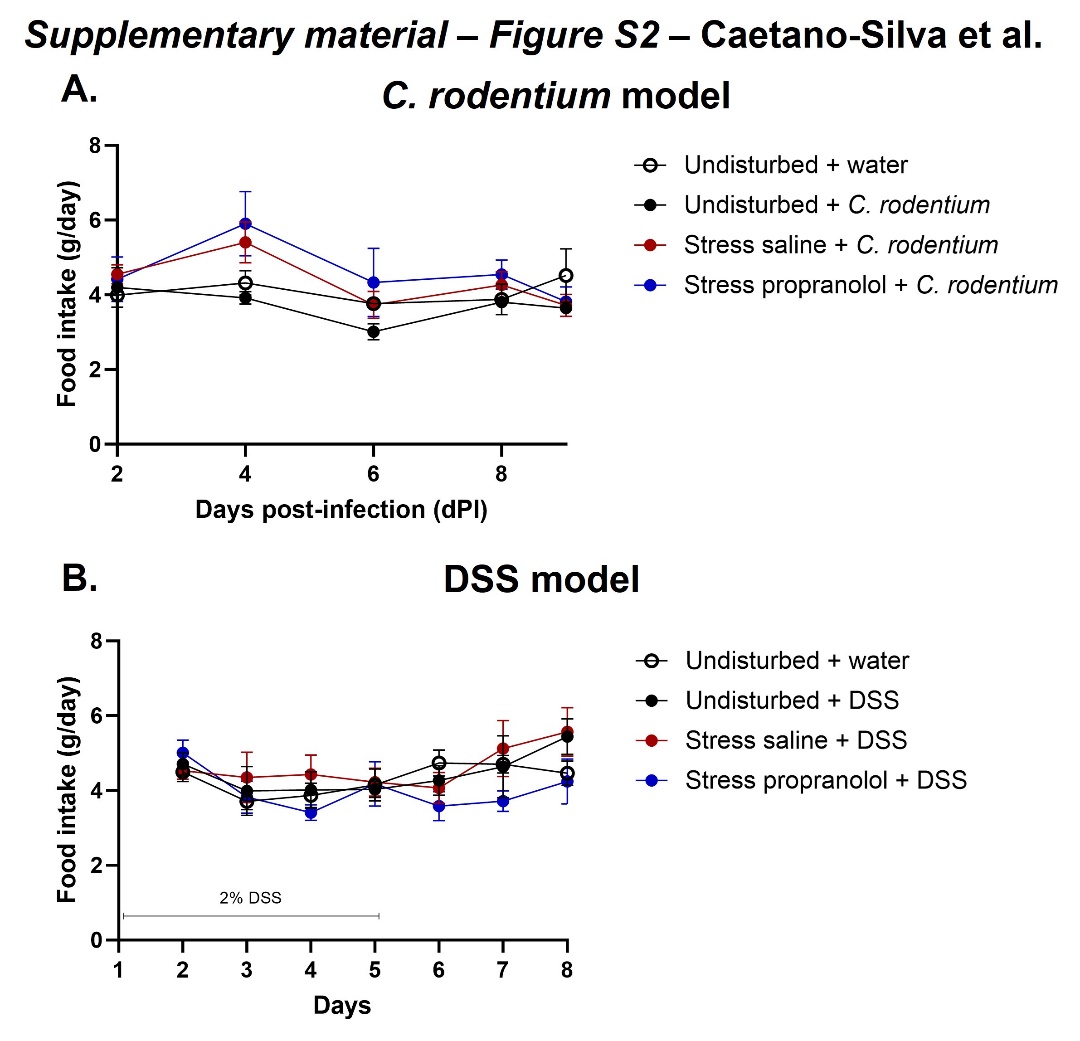


**Supplementary Figure S4. Colonic gene expression during *C. rodentium* infection with stress and β-adrenergic blockade. (A)** Timeline for *C. rodentium* infection combined with SDR paradigm. dPI = days post-infection. Mice received β-AR antagonist (propranolol; 10 mg/kg, i.p.) or saline before each stress session. Mice were oral challenged with 3 x 10^7^ CFU of *C. rodentium* at Day 4 of SDR paradigm (d0PI). **(B)** Fold change gene expression of colonic ROS/RNS-related enzymes (*Duox2, Duoxa2, Nos2*) and chemokine *Ccl2*, relative to control uninfected group. Values were log-transformed prior to analysis. One-way ANOVA was used, followed by post hoc comparisons for selected pairs: Control *C. rodentium* vs. Stress *C. rodentium* and Stress *C. rodentium* vs. Stress Propranolol *C. rodentium*. An unpaired t-test compared Control uninfected and Control *C. rodentium*. Data are presented as mean ± SEM, with *p* < 0.05considered statistically significant. n=4-6/group.


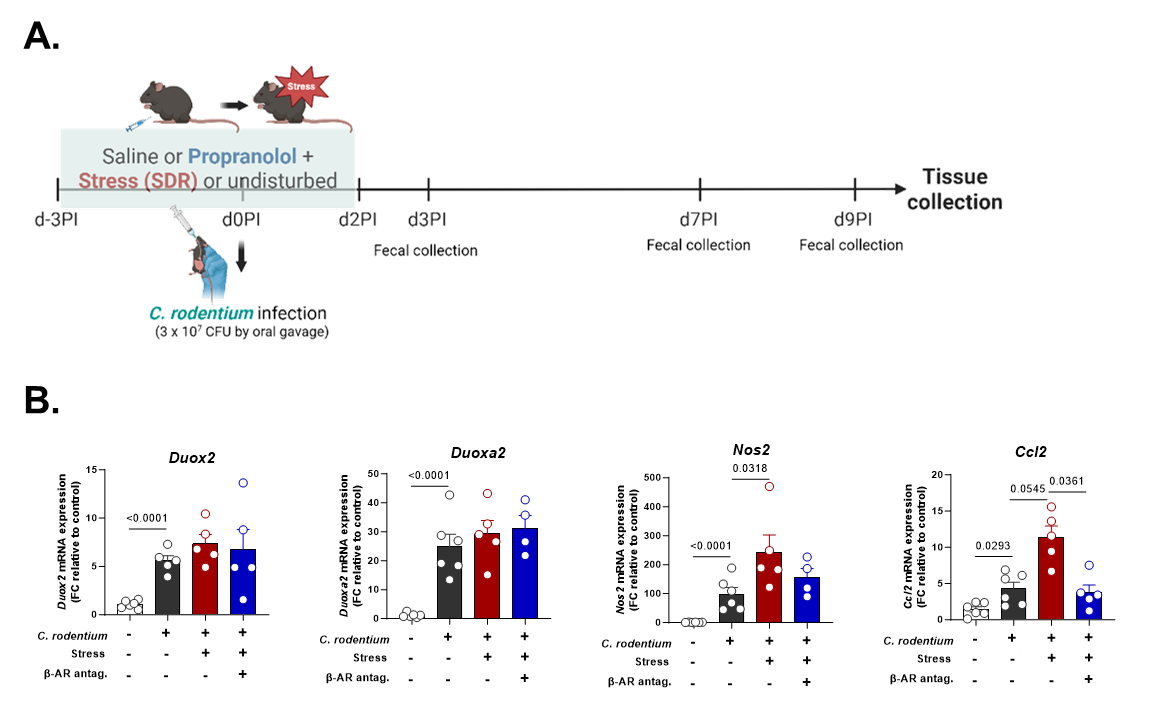


**Supplementary Figure S5. β2-adrenergic blockade with zenidolol modulates stress-exacerbated C. rodentium colitis. (A)** Timeline for *C. rodentium*-induced colitis + SDR paradigm with β2-AR antagonist (zenidolol) treatment. dPI = days post-infection. Mice were orally challenged with 3 × 10⁷ CFU of *C. rodentium* at Day 4 of SDR paradigm (d0PI); **(B)** Body weight (BW) changes as % of baseline at d9PI; **(C)** Colon length; **(D)** Total histopathology score of distal colon sections stained with H&E; **(E)** Representative H&E images from stressed infected mice with or without zenidolol treatment. Scale bars = 10 μm. Data are presented as mean ± SEM relative to infected unstressed mice. Unpaired t test compared both groups, with p < 0.05 considered statistically significant. n = 7-8/group.


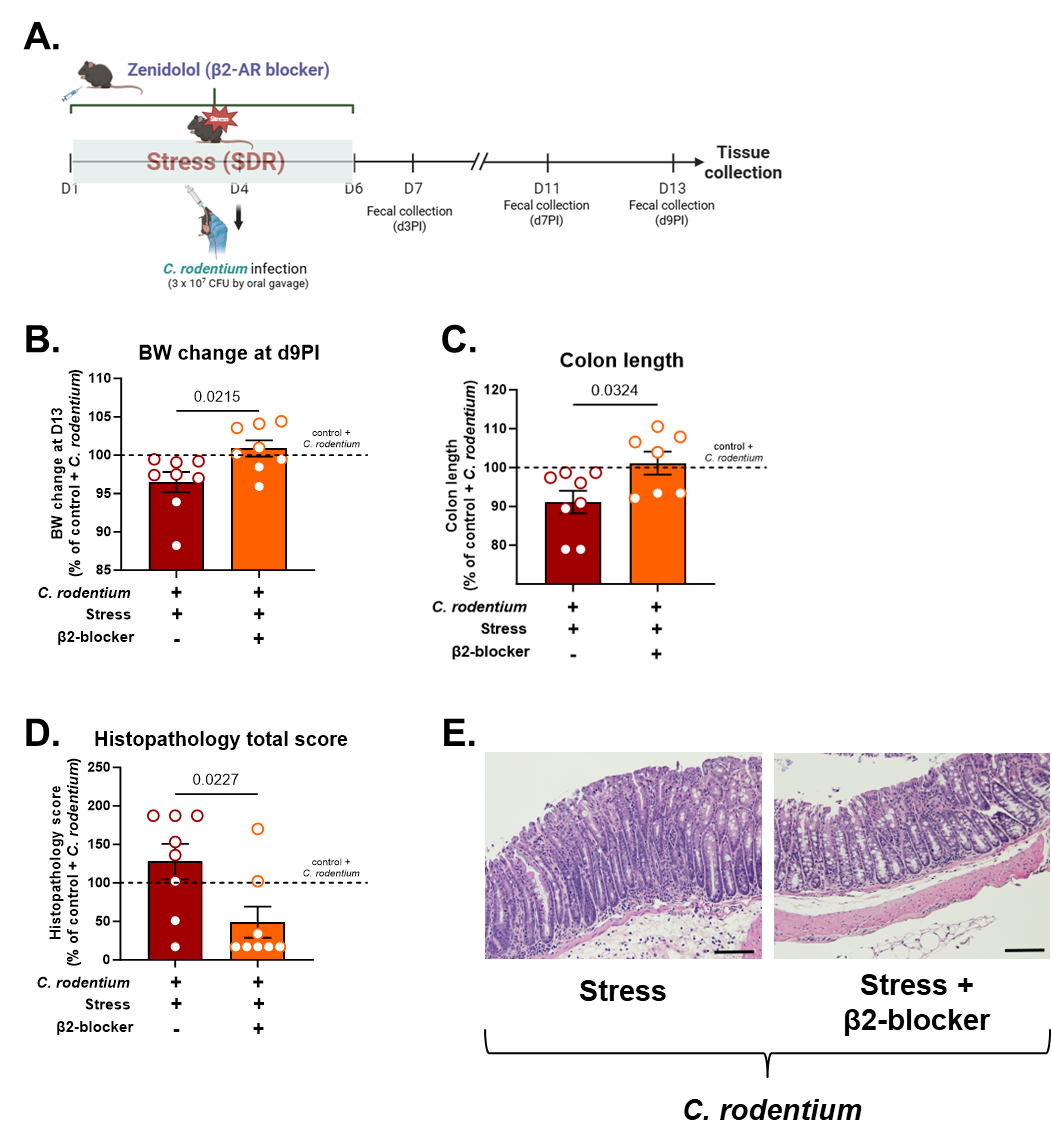


**Supplementary Figure S6. Apocynin reduces stress-exacerbated colitis histopathology during *C. rodentium* infection. (A)** Timeline for *C. rodentium* infection combined with SDR paradigm and apocynin treatment. dPI = days post-infection. **(B)** Total histopathology score of distal colon sections stained with H&E at d9PI. **(C)** Representative H&E images of distal colon from stressed infected mice with or without apocynin treatment. Scale bars = 100 μm. Data are presented as mean ± SEM. Statistical analysis was performed by unpaired *t*-tests comparing stressed + *C. rodentium* vs. stressed + *C. rodentium* + apocynin groups. *p* < 0.05 was considered statistically significant. *n* = 5–6/group.


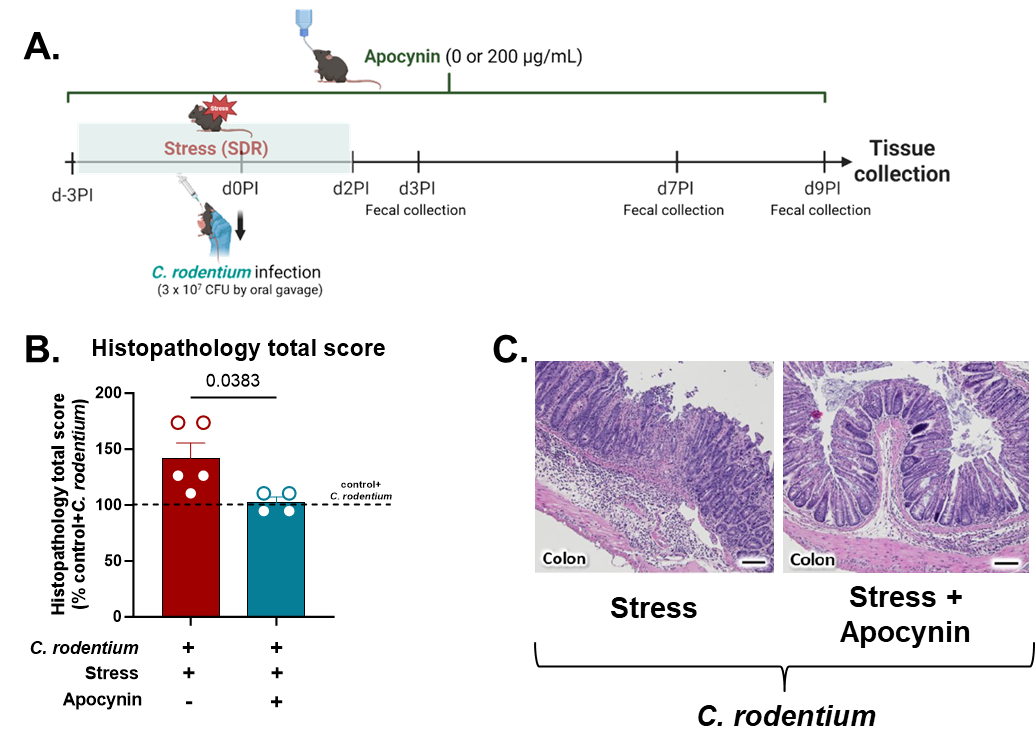


**Supplementary Figure S7. Transient NADPH oxidase inhibition during the SDR period recapitulates the protective effects of continuous apocynin treatment. (A–B)** Experimental timeline for *C. rodentium*– and DSS-induced colitis combined with SDR paradigm, with apocynin administered only during SDR cycles (Day 1–6). dPI = days post-infection. **(C–D)** Body-weight (BW) change at **(C)** d9PI/D13 relative to infected unstressed controls and at **(D)** D15 relative to DSS-treated unstressed mice. **(E-F)** Colon length as a percentage of **(E)** infected and **(F)** DSS-treated unstressed controls. **(G)** Disease activity index (DAI) at D7 relative to DSS-treated mice. Data are presented as mean ± SEM; p < 0.05 was considered statistically significant. n = 5–6 per group.


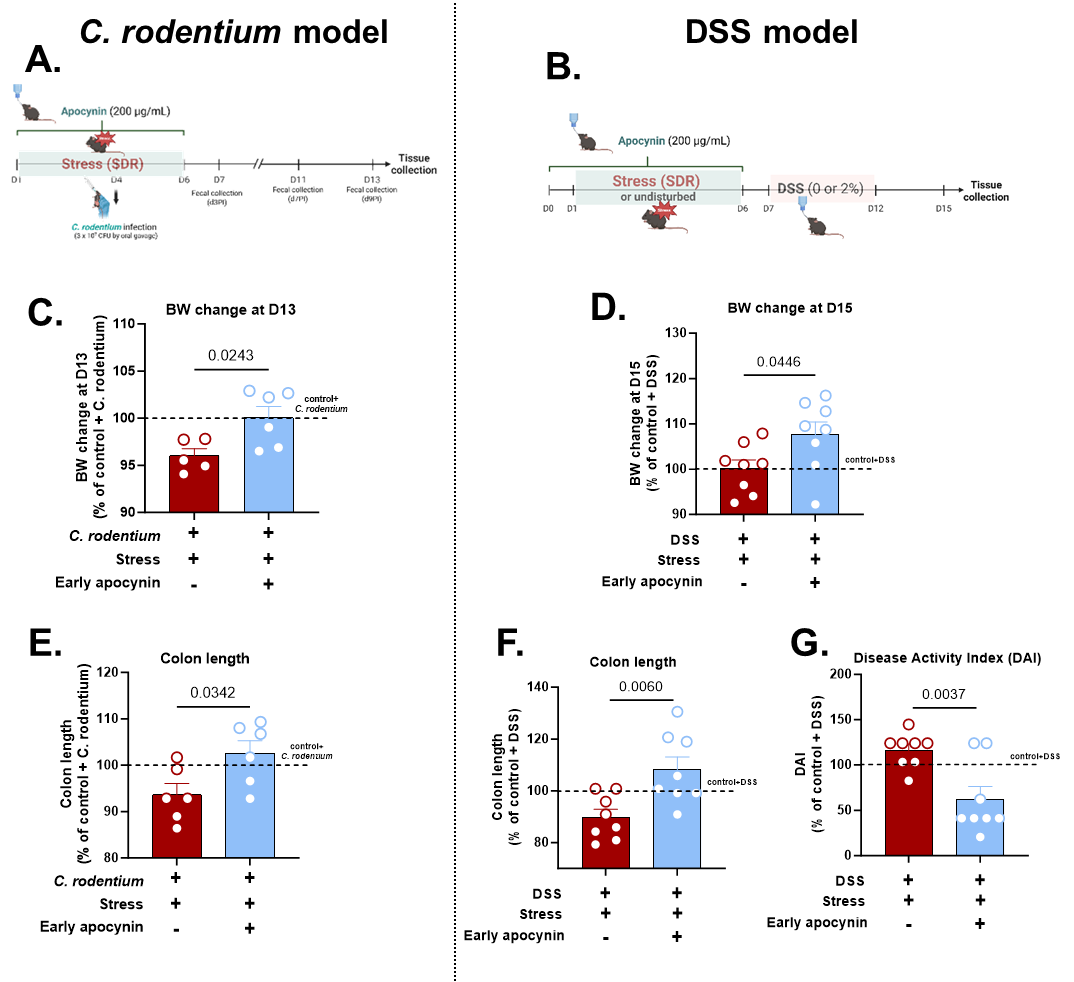


**Supplementary Figure S8. N-acetylcysteine (NAC) fails to protect against stress-exacerbated colitis. (A)** Timeline for *C. rodentium*-induced colitis + SDR paradigm with NAC treatment (40 mM) in drinking water. dPI = days post-infection. Mice were orally challenged with 3 × 10⁷ CFU of *C. rodentium* at Day 4 of SDR paradigm (d0PI). **(B-C)** Body weight (BW) change as % of baseline at **(B)** d3PI (after SDR) and **(C)** d9PI. **(C)** Colon length. Data are presented as mean ± SEM relative to infected unstressed mice. Unpaired t test compared both groups, with p < 0.05 considered statistically significant. n = 5-6/group.


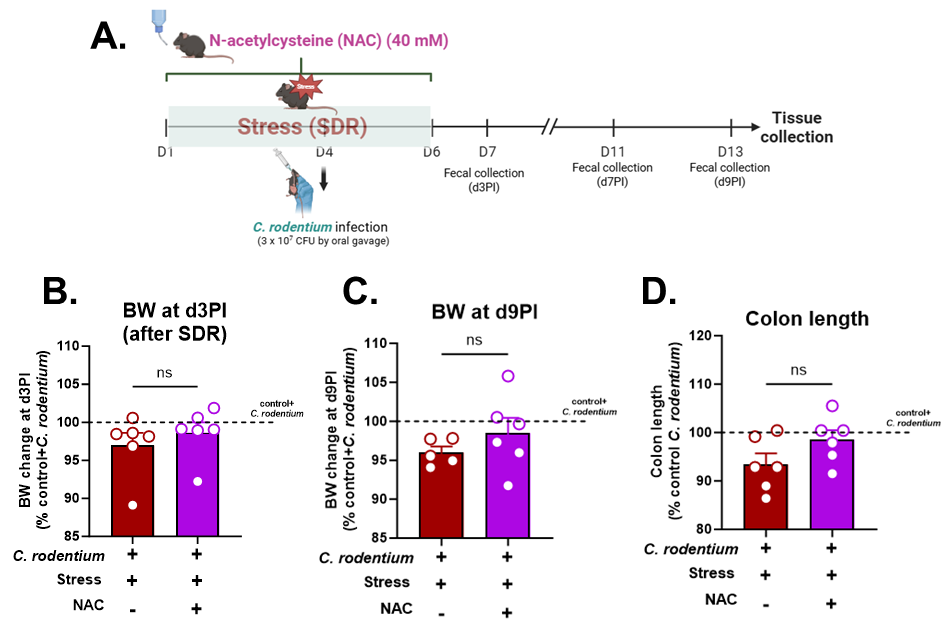

Supplement: Supplementary Data [file NIHMS2143753-supplement-Supplementary_Data.docx]
